# Integrative Multiomics Analysis Identifies a Novel Gene Signature That Predicts Chemotherapy Resistance and Poor Survival in Osteosarcoma

**DOI:** 10.1155/humu/8885469

**Published:** 2026-05-30

**Authors:** Xiyu Yang, Xiaoming Lu, Zhiqiang Gao, Wentao Lv, Shenghuang Shen, Sijia Liu, Zetong Li, Ruixuan Liu, Xinyao Wang, Yao Shen, Danlei Yu, Xuanhong Jin, Haowei Wang, Jianfei Tang, Zhuoran Tang

**Affiliations:** ^1^ Department of Pathology, Medical School of Nantong University, Nantong, China, ntu.edu.cn; ^2^ Department of Orthopedic Oncology, Shanghai General Hospital, School of Medicine, Shanghai Jiao Tong University, Shanghai, China, sjtu.edu.cn; ^3^ Affiliated Qidong Hospital of Nantong University, Qidong People’s Hospital, Qidong Liver Cancer Institute, Qidong, China; ^4^ Department of Joint Surgery, Shanghai East Hospital, School of Medicine, Tongji University, Shanghai, China, tongji.edu.cn; ^5^ Department of Medical Oncology, Shanghai East Hospital, Tongji University School of Medicine, Shanghai, China, tongji.edu.cn; ^6^ Department of Orthopedic Surgery, Shanghai Sixth People’s Hospital Affiliated to Shanghai Jiao Tong University School of Medicine, Shanghai, China, sjtu.edu.cn

**Keywords:** chemotherapy resistance, gene signature, multiomics, osteosarcoma, tumor microenvironment

## Abstract

Chemotherapy resistance is the primary barrier to improving survival in osteosarcoma (OS), yet reliable predictive biomarkers remain limited. Here, we developed and validated a 13‐gene signature via weighted gene coexpression network analysis (WGCNA) of chemotherapy‐resistant and sensitive OS transcriptomes. The risk model demonstrated robust prognostic value across four independent cohorts (TARGET‐OS, GSE21257, GSE16091, and GSE39055). Multiscale analysis revealed that high‐risk tumors exhibit proliferative hyperactivation, genomic instability with elevated TMB, and a paradoxical immune‐cold phenotype despite the high mutational load. Single‐cell and spatial transcriptomics demonstrated that high‐risk malignant cells display dedifferentiated, stem‐like properties, preferentially localize to hypoxic necrotic peripheries, and engage in extensive tumor–stromal crosstalk. Drug sensitivity predictions confirmed resistance to first‐line chemotherapy agents. Collectively, these findings establish a clinically actionable 13‐gene biomarker and provide a mechanistic framework linking transcriptional profiles to chemoresistance biology, exposing novel therapeutic vulnerabilities in OS.

## 1. Introduction

Osteosarcoma (OS) is a highly malignant tumor that originates from primitive mesenchymal stem cells. It is also the most common primary bone malignancy in children and adolescents [1]. Its characteristic is that tumor cells directly produce immature bone‐like tissue, and it has a high degree of invasiveness and an early tendency for hematogenous metastasis [2, 3]. The lungs are the most common site of metastasis. Over the past few decades, the combined treatment strategy of neoadjuvant chemotherapy based on methotrexate, doxorubicin, and cisplatin (MAP regimen) and extensive surgical resection has become the standard first‐line treatment for OS [4, 5]. This multimodal treatment has significantly increased the 5‐year overall survival rate of patients from less than 20% to 60%–70% [6, 7]. However, this survival benefit has entered a clearly plateaued stage in recent years. Clinical data show that approximately 30%–40% of patients exhibit primary or acquired resistance to the standard chemotherapy regimen [8, 9]. These patients are highly prone to local recurrence or distant metastasis, and their 5‐year survival rate drops sharply to below 20%. Chemotherapy resistance is not only the fundamental cause of treatment failure but also the insurmountable bottleneck in improving the prognosis of OS patients at present [10, 11]. Therefore, an in‐depth analysis of the molecular mechanisms underlying chemotherapy resistance in OS and the search for biomarkers that can predict the chemotherapy response early and guide stratified treatment are the key issues that need to be urgently addressed in clinical practice at present.

OS exhibits significant tumor heterogeneity and a complex genomic profile, including frequent somatic copy number variations and structural variations. This makes the traditional anatomical‐based clinical staging system severely limited in predicting individualized drug responses and survival outcomes [12, 13]. In recent years, the rapid development of high‐throughput sequencing technology has provided a new opportunity for a comprehensive analysis of the molecular characteristics of tumors [14, 15]. The multiomics integration analysis, which encompasses genomics, transcriptomics, and epigenomics, is gradually emerging as a powerful tool for revealing the complex biological behaviors of tumors. Although researchers have identified various gene characteristics related to the prognosis of OS through transcriptome sequencing data, most of the existing studies are limited to the expression level of individual genes and lack the systematic exploration of specific coexpression networks related to chemotherapy resistance [16, 17]. Furthermore, there are few studies that integrate transcriptome characteristics with genomic variations to comprehensively explain the molecular driving forces behind drug resistance. Currently, the existing prognostic models still face challenges in terms of insufficient robustness in clinical applications and a lack of specific interpretations of the mechanisms of chemotherapy resistance [18].

In addition to the genetic variations of the tumor cells themselves, the tumor microenvironment (TME) and its immune status play a crucial role in the progression and treatment response of OS. In recent years, immunotherapy based on immune checkpoint inhibitors, such as PD‐1/PD‐L1 antibodies, has achieved significant breakthroughs in various solid tumors. However, the clinical benefits of immunotherapy in OS remain limited [19, 20]. This is mainly attributed to the unique immune microenvironment characteristics of OS. It is generally regarded as an immune‐cold tumor, characterized by insufficient infiltration of effector T cells, while regulatory T cells (Tregs) and M2‐type macrophages, among others, which are inhibitory cells, are abundantly present, resulting in severe immune escape. Increasing evidence indicates that there is a close interaction between chemotherapy resistance and the immunosuppressive microenvironment [21]. Tumors that fail to respond to chemotherapy often exhibit a stronger ability to evade the immune system. Therefore, relying solely on conventional bulk transcriptome sequencing (bulk RNA‐seq) often masks the complex heterogeneity of immune cell subpopulations within the TME [22, 23]. The breakthrough application of single‐cell RNA sequencing (scRNA‐seq) technology enables us to analyze the tumor ecosystem at the single‐cell level and precisely identify specific cell subpopulations and their communication networks, thereby revealing the common mechanism of chemotherapy resistance and immune escape [24, 25].

Our study aims to construct a robust prognostic model based on key genes related to chemotherapy resistance by integrating the transcriptome sequencing data of our center with multiomics resources from public databases. We used weighted gene coexpression network analysis (WGCNA) to screen out core genes closely related to resistance and constructed a 13‐gene signature. Subsequently, we verified the prognostic value of this model in multiple independent cohorts and combined somatic mutation, drug sensitivity, and immune infiltration analyses to comprehensively explore the biological basis and microenvironment characteristics behind the high‐risk score. Finally, we introduced single‐cell and spatial sequencing data to deeply investigate the communication and interaction between tumor cells and microenvironment cells, with the aim of providing new targets and a theoretical basis for the precise treatment of OS.

## 2. Materials and Methods

### 2.1. Sample Collection

All patients participating in this study or their legal guardians signed written informed consent forms before the biopsy. This study used bone cancer patient samples from our center. All samples were confirmed by pathology to be from bone cancer patients, and the treatment was chemotherapy. The collected bone cancer tissue samples were divided into the chemotherapy‐resistant group and the chemotherapy‐sensitive group based on the clinical chemotherapy response, according to the RECIST standard.

### 2.2. Sample Processing and RNA Extraction

A total of 11 tumor samples from OS patients were collected. All the samples were immediately frozen and stored at −80°C. Total RNA extraction was performed using TRIzol reagent (Invitrogen, United States), following the manufacturer’s instructions. The quality of the RNA was assessed using NanoDrop 2000 (Thermo Fisher Scientific, United States), ensuring an A260/A280 value between 1.8 and 2.0, with an RNA concentration of no less than 100 ng/*μ*L. The extracted RNA was used for subsequent transcriptome sequencing analysis.

### 2.3. Transcriptome Sequencing and Data Preprocessing

The total RNA of 11 samples was subjected to high‐throughput transcriptome sequencing using the Illumina NovaSeq 6000 platform (Illumina, United States). The raw sequencing data were quality‐controlled using FastQC (Version 0.11.9), and low‐quality reads were removed. Subsequently, the raw data were cleaned using Trimmomatic (Version 0.39) for the removal of adapter sequences and low‐quality sequences. The cleaned data were then aligned using HISAT2 (Version 2.2.1), with the reference genome being the GRCh38 version [26–32]. Finally, the gene expression matrix for each sample was obtained.

### 2.4. Analysis of Differentially Expressed Genes (DEGs)

DESeq2 (Version 1.30.0) was used to conduct differential gene expression analysis (DEA) on the transcriptome data. Genes with significant differences between the chemotherapy‐resistant group and the sensitive group were selected based on the expression level differences of the genes. We used the following criteria to screen the DEGs: the absolute value of the log2 fold change (log2FC) > 1 and *p* < 0.05. A volcano plot was used to display the distribution of the DEGs.

### 2.5. Gene Coexpression Network Analysis and Prognostic Model Construction

To identify the key genes related to chemotherapy resistance and prognosis, WGCNA (v1.68) was employed. In WGCNA, 1752 DEGs were selected and classified into six modules based on their gene expression patterns. By calculating the correlations between each module and clinical characteristics, the gray module significantly related to prognosis was screened out. This module contains 13 key genes. Based on these 13 genes, a prognostic risk score model, the 13‐gene signature, was constructed. Using the Cox regression model, the risk score of each patient was calculated, and the relationship between the risk score and the patient’s survival period was evaluated through Kaplan–Meier (K‐M) survival analysis (log‐rank test) [33, 34].

### 2.6. Acquisition and Processing of Public Datasets

We obtained the OS cohort data from multiple public databases. For public datasets, we downloaded OS transcriptome data and corresponding clinical survival information from the TARGET database (https://ocg.cancer.gov/programs/target) and the GEO database, including TARGET‐OS, GSE21257, GSE16091, and GSE39055 [35–41]. Subsequently, we combined these three datasets from GEO and used the removeBatchEffect function from the linear model (limma) in the R software package to remove the batch effect. We visualized the distribution of samples before and after correction using principal component analysis (PCA) to ensure that the batch effect was effectively removed.

### 2.7. Functional Enrichment Analysis

Enrichment analysis of GO and KEGG pathways was conducted for the DEGs and the genes related to the 13‐gene signature. The GO and KEGG pathway analyses were performed using the clusterProfiler package (Version 3.18.1).

### 2.8. Genome Mutation Analysis

We used the R package maftools to analyze somatic mutation data (in MAF format). We compared the tumor mutation burden (TMB) between the high‐risk and low‐risk groups, and we drew a waterfall plot to display the frequently mutated genes and mutation types in each group.

### 2.9. Drug Sensitivity Prediction and Analysis of the Immune Microenvironment

Based on the GDSC (Genomics of Drug Sensitivity in Cancer) database, the R package pRRophetic was used to predict the half‐maximal inhibitory concentration (IC50) of various samples for commonly used chemotherapy drugs [42]. At the same time, the expression differences of drug target genes between the high‐risk and low‐risk groups were analyzed. The CIBERSORT algorithm was used to calculate the infiltration ratios of 22 types of immune cells in the TME. The TIDE (Tumor Immune Dysfunction and Exclusion) algorithm was used to predict the immune escape score of patients and the possibility of responding to immune checkpoint blockade (ICB) therapy online.

### 2.10. scRNA‐seq Data Processing

scRNA‐seq data preprocessing and downstream analyses were performed using the Seurat package (Version 4.4.0) in R, integrating publicly available OS datasets GSE162454 and GSE152048 [14, 43]. Genes expressed in fewer than 100 cells, as well as mitochondrial, ribosomal protein, and noncoding RNA genes, were excluded, and cells were filtered based on nFeature_RNA, mitochondrial percentage, hemoglobin percentage, and nCount_RNA [44]. Doublet cells were removed using scDblFinder (v1.18.0), and ambient RNA contamination was corrected using decontX, with cells showing contamination scores > 0.2 excluded. The filtered matrices were normalized using the LogNormalize method, 3000 highly variable genes were identified, and batch effects were corrected using the Harmony algorithm (v0.1.0). t‐SNE dimensionality reduction was performed on Harmony‐corrected embeddings using the top 15 principal components, followed by unsupervised clustering at multiple resolutions. Cluster‐specific marker genes were identified using FindAllMarkers and genesorteR, and cell populations were annotated based on canonical markers from CellMarker2.0 and previous publications, identifying OS cells, tumor‐associated macrophages (TAMs), fibroblasts, T/NK cells, B cells, osteoclasts, monocytes, endothelial cells, pericytes, and proliferating cell populations [14].

### 2.11. scRNA‐seq Data Analysis

The 13‐gene signature was mapped to single‐cell data using the UCell R package. The comprehensive risk score was calculated by subtracting the downregulated gene score from the upregulated gene score associated with chemotherapy resistance. Cells were classified as high risk or low risk based on the mean score threshold of OS cells. To investigate key biological processes in OS, we obtained 50 hallmark gene sets from the MSigDB database and 14 cancer cell functional state gene sets from the CancerSEA database (http://biocc.hrbmu.edu.cn/CancerSEA/). Comprehensive pathway enrichment analysis was performed using six gene set scoring methods (AUCell, AddModuleScore, UCell, singscore, ssGSEA, and zscore), with results integrated via robust rank aggregation (RRA) using the irGSEA and RobustRankAggreg R packages (significance threshold: RRA score < 0.05). DEA between risk groups was conducted using Libra (v1.0.0) with the Wilcoxon rank‐sum test, identifying genes with |log2*F* *C*| > 0.25 and adjusted *p* < 0.05 as significantly differentially expressed. Cell differentiation trajectories were inferred using Monocle2, and intercellular communication networks were analyzed with CellChat (v1.1.3) to identify interactions between tumor cells and microenvironment cells [45].

### 2.12. Validation Using Spatial Transcriptomic Data

We obtained publicly available spatial transcriptomic data from a prior OS investigation (https://codeocean.com/capsule/9535428/tree/v1), which included both spatial gene expression profiles and corresponding H&E histological sections. Data processing was conducted through the Seurat framework, applying SCTransform for normalization and employing FindIntegrationAnchors for cross‐sample integration, consistent with our scRNA‐seq analytical pipeline. Spatial clustering was executed via the FindVariableFeatures, FindNeighbors, and FindClusters functions, while cluster‐defining genes were determined using FindAllMarkers to facilitate cellular identity assignment [28, 46–50]. To transfer cell type labels from our scRNA‐seq reference onto the spatial dataset, we implemented the RCTD deconvolution algorithm, enabling precise cell type attribution to individual spatial locations and enhanced interpretation of spatially resolved expression patterns [51–55]. We subsequently computed the 13‐gene signature score through UCell and visualized its spatial distribution to identify chemoresistant cellular niches. To confirm the predictive robustness of our signature, we used the GDSC database combined with the pRRophetic package to estimate IC50 values of chemotherapeutic agents across spatially defined risk spots.

### 2.13. Statistical Analysis

All statistical analyses were conducted using R software Version 4.2.0. Comparisons between the two groups were performed using the Wilcoxon rank‐sum test or *t*‐test; comparisons among multiple groups were conducted using the Kruskal–Wallis test. Correlation analysis used the Spearman or Pearson correlation coefficients. K‐M survival analysis was performed using the log‐rank test. All statistical tests were two‐sided, and a *p* value < 0.05 was considered statistically significant.

## 3. Results

### 3.1. Identification of Chemotherapy Resistance–Associated Candidate Genes

**Figure 1:**
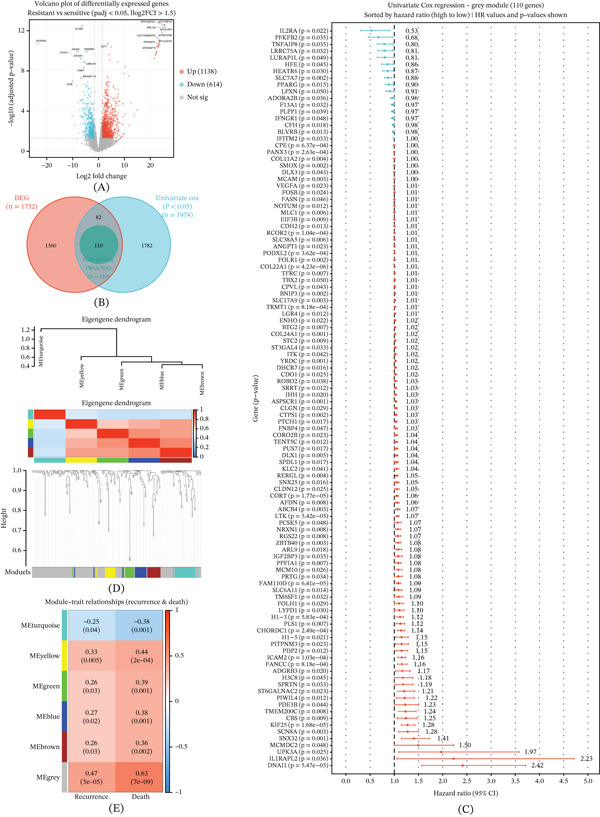
Identification of chemotherapy resistance–associated candidate genes through transcriptome analysis and weighted gene coexpression network analysis (WGCNA). (A) The volcano plot displays differentially expressed genes between chemotherapy‐resistant and sensitive osteosarcoma samples, where red dots indicate upregulated genes, blue dots indicate downregulated genes, and gray dots represent genes without significant differences. (B) The Venn diagram illustrates the intersection of 1752 differentially expressed genes from our institutional cohort and 1974 prognostic‐related genes from public databases, resulting in 110 overlapping genes that are associated with both chemotherapy resistance and patient prognosis. (C) The forest plot presents the hazard ratios and statistical significance of these 110 overlapping genes as determined by univariate Cox regression analysis. (D) The WGCNA dendrogram and module–trait relationship heatmap. (E) The gray module shows the strongest positive correlation with both recurrence (*r* = 0.47) and death (*r* = 0.63) and contains the final 13 key genes used for signature construction.

To explore the key molecular mechanisms underlying chemotherapy resistance in OS, we collected OS samples from our center (*n* = 11) and conducted transcriptome sequencing analysis. Based on the chemotherapy responsiveness, the samples were divided into the resistant group and the sensitive group. DEA revealed significant differences in gene expression between the two groups, and a total of 1752 DEGs were identified. The expression differences of these genes were visually presented in a volcano plot (Figure 1A), where red dots represent upregulated genes, blue dots represent downregulated genes, and gray dots represent genes with no significant differences.

To further select the candidate genes with clinical prognostic value, we took the intersection of these differential genes with the 1974 prognostic‐related genes screened out through univariate Cox regression analysis from the public database. Eventually, we obtained 110 overlapping genes that were both associated with chemotherapy resistance and significantly correlated with the patients’ prognosis (Figure 1B). Subsequently, we used univariate Cox regression analysis to evaluate the prognostic value of these genes, and we presented the hazard ratios (HRs) and significance of these 110 genes in a forest plot (Figure 1C).

To extract the core gene set from these 110 genes, we employed WGCNA (Figure 1D). The clustering results indicated that these genes were classified into six modules. By analyzing the correlation between the modules and prognosis, we found that the gray module showed the strongest positive correlation with the recurrence (*r* = 0.47) and death (*r* = 0.63) of OS (Figure 1E). This module contained 13 key genes. Based on this, we constructed a prognostic risk score model consisting of 13 genes (13‐gene signature).

### 3.2. Validation of the 13‐Gene Signature for Prognostic Prediction Across Multiple Cohorts

**Figure 2:**
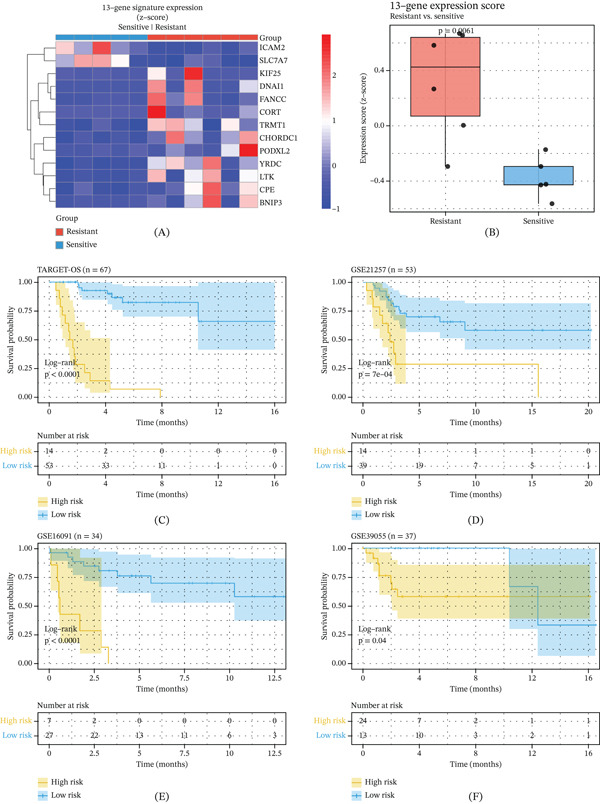
Validation of the 13‐gene signature for prognostic prediction across multiple independent cohorts. (A) The heatmap illustrates the expression patterns of the 13 signature genes across chemotherapy‐resistant and sensitive groups from our institutional cohort. (B) The box plot demonstrates that risk scores calculated from the 13‐gene signature are significantly higher in the resistant group compared to the sensitive group (*p* = 0.0061). (C) The Kaplan–Meier survival curve from the TARGET‐OS cohort shows that patients in the high‐risk group have significantly poorer overall survival than those in the low‐risk group (log‐rank *p* < 0.0001). (D) The Kaplan–Meier survival curve from the GSE21257 cohort confirms that high‐risk scores are significantly associated with worse prognosis (log‐rank *p* = 7*e* − 04). (E) The Kaplan–Meier survival curve from the GSE16091 cohort demonstrates consistent results with significantly reduced survival in high‐risk patients. (F) The Kaplan–Meier survival curve from the GSE39055 cohort further validates the robust prognostic value of the 13‐gene signature across independent datasets.

The heatmap showed that these 13 genes generally exhibited a high expression pattern in the resistant group (Figure 2A). More importantly, the risk score calculated based on this model was significantly higher in the resistant group than in the sensitive group (*p* = 0.0061), suggesting that this score can effectively distinguish the chemotherapy responsiveness of patients (Figure 2B). To verify the robustness and universality of this risk scoring system, we conducted validations in multiple independent public datasets (TARGET‐OS, GSE21257, GSE16091, and GSE39055) after removing the batch effect. We divided the patients into high‐risk and low‐risk groups based on the median value of the risk score. In the TARGET‐OS cohort (*n* = 67), the K‐M survival analysis showed that the overall survival of patients in the high‐risk group was significantly lower than that in the low‐risk group (log‐rank *p* < 0.0001, Figure 2C). Similarly, in the GSE21257 cohort (*n* = 53), the high‐risk score was also significantly associated with a poorer prognosis (log‐rank *p* = 7*e* − 04, Figure 2D). Moreover, we also observed a completely consistent trend in the GSE16091 and GSE39055 cohorts (Figure 2E,F). These results strongly confirm that our 13‐gene signature is a robust and efficient prognostic predictor for OS.

### 3.3. Distinct Biological Functions, Genomic Mutation Landscape, and Immune Features Between Different Risk Subgroups

To elucidate the potential biological mechanisms underlying the adverse prognosis and chemotherapy resistance caused by high‐risk scores, we conducted a functional enrichment analysis on the sequencing data of samples from the high‐risk and low‐risk groups. The GO analysis revealed that the genes upregulated in the high‐risk group were mainly enriched in processes related to the cell cycle, such as chromosome segregation, organelle fission, and DNA replication (Figure 3A). The KEGG pathway analysis further confirmed that the cell cycle, p53 signaling pathway, and cytokine–receptor interaction were significantly activated in the high‐risk group (Figure 3B). These results suggest that tumor cells in the high‐risk group have more vigorous proliferative ability and more active cell division activities.

Figure 3Distinct biological functions, genomic mutation landscape, and immune features between risk subgroups. (A) The Gene Ontology enrichment analysis reveals that genes upregulated in the high‐risk group are significantly enriched in cell cycle–related biological processes, including chromosome segregation, organelle fission, and DNA replication. (B) The KEGG pathway analysis further confirms that the cell cycle, p53 signaling pathway, and cytokine–receptor interaction are significantly activated in high‐risk tumors. (C) The oncoplot waterfall diagram displays the somatic mutation landscape in the low‐risk group, showing the distribution of frequently mutated genes and mutation types. (D) The oncoplot waterfall diagram presents the somatic mutation landscape in the high‐risk group, where TP53 and MUC16 exhibit higher mutation frequencies compared to the low‐risk group. (E) The box plot compares tumor mutation burden (TMB) between risk groups and demonstrates that TMB is significantly elevated in high‐risk patients. (F) The expression analysis of drug target genes reveals significant differences between the high‐risk and low‐risk groups for key targets of etoposide, doxorubicin, and methotrexate. (G) The drug sensitivity prediction analysis based on the GDSC database shows that the high‐risk group exhibits potential resistance to multiple first‐line chemotherapy drugs. (H) The comparison of IC50 values confirms that high‐risk patients display markedly reduced sensitivity to standard chemotherapeutic agents. (I) The TIDE algorithm analysis indicates that patients in the high‐risk group have significantly increased immune escape scores, suggesting poor response to immune checkpoint blockade therapy. (J) The CIBERSORT analysis of immune cell infiltration patterns reveals that the high‐risk group has a unique immune cell composition that contributes to an immunosuppressive microenvironment and cold tumor phenotype.

**Figure:**
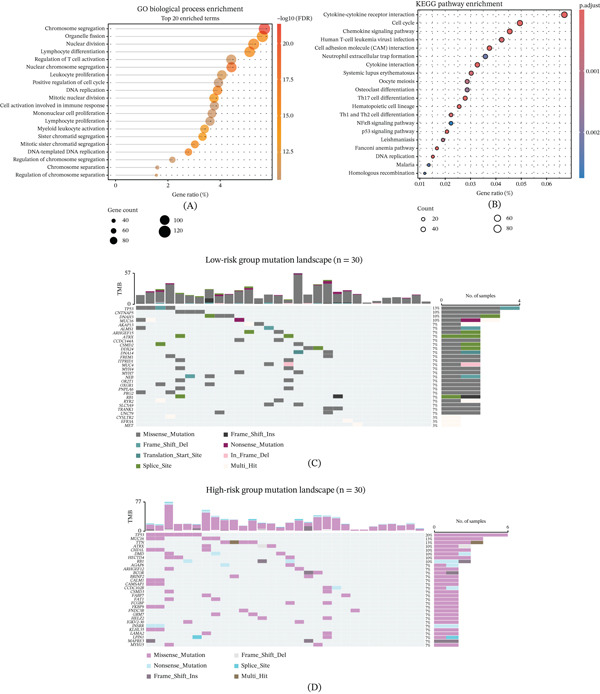

**Figure:**
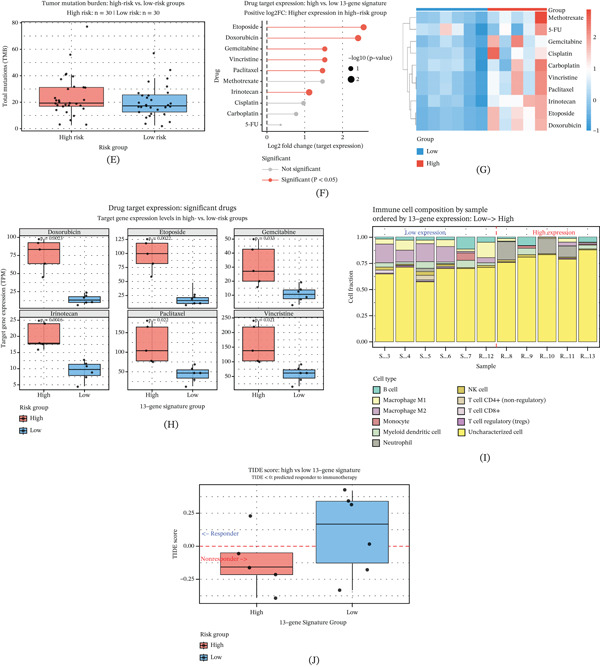

At the genomic level, the mutation waterfall plot (Figure 3C,D) presents the specific mutation profile. Genes such as TP53 and MUC16 exhibit higher mutation frequencies in the high‐risk group. This specific mutation pattern may drive the malignant progression of tumors and their resistance to treatment. Additionally, we analyzed the TMB. The results showed that the TMB in the high‐risk group was significantly higher than that in the low‐risk group (Figure 3E), which typically indicates higher genomic instability, suggesting that the high‐risk group may have a higher mutation rate, leading to tumor resistance and malignant progression.

Given the chemotherapy resistance characteristics exhibited by the high‐risk group, we further evaluated the potential of this model in guiding clinical medication. We analyzed the expression of genes targeting common chemotherapy drugs and found that multiple key drug targets, such as those for chemotherapy drugs like etoposide, doxorubicin, and methotrexate, showed significant differences in expression between the high‐risk and low‐risk groups (Figure 3F). By comparing the risk scores with the drug sensitivity database, we discovered that the high‐risk group exhibited potential resistance to multiple first‐line chemotherapy drugs (Figure 3G,H). This result is consistent with our observations of chemotherapy resistance in clinical practice, suggesting that this model may be helpful in predicting patients’ resistance to chemotherapy drugs. Furthermore, for the prediction of the response to immunotherapy, we evaluated it using the TIDE algorithm. The results showed that the TIDE score of patients in the high‐risk group was significantly increased (Figure 3I). A higher TIDE score typically indicates the presence of an immune escape mechanism in the tumor and poor response to ICB. Combined with the analysis of immune cell infiltration (Figure 3J), we found that the high‐risk group had a unique immune cell composition pattern, which might jointly construct an inhibitory immune microenvironment, leading to the cold tumor phenotype, thereby limiting the efficacy of immunotherapy.

### 3.4. Single‐Cell Transcriptomic Analysis of Cellular Heterogeneity and Functional Characteristics of the 13‐Gene Signature

**Figure 4:**
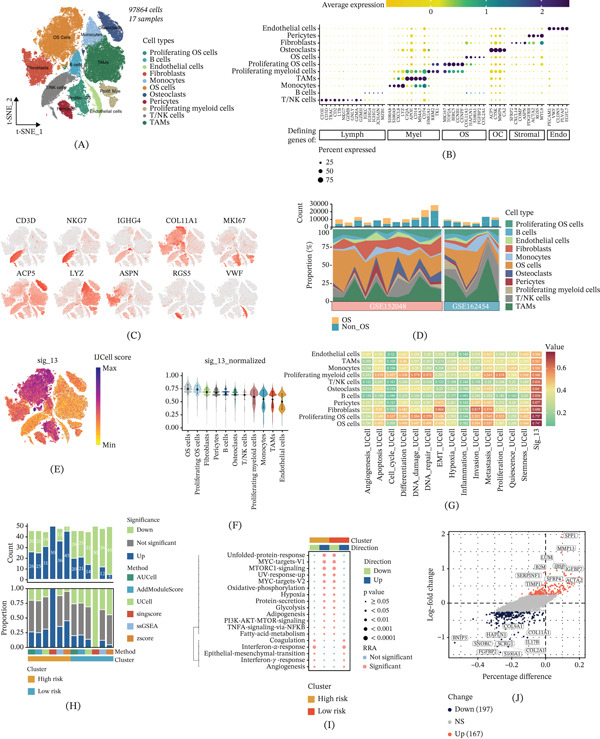
Single‐cell transcriptomic analysis of cellular heterogeneity and functional characteristics of the 13‐gene signature. (A) The t‐SNE plot displays the major cell populations identified from 97,864 cells across 17 osteosarcoma (OS) samples, including OS cells, proliferating OS cells, osteoblasts, fibroblasts, monocytes, myeloid cells, T/NK cells, TAMs, B cells, endothelial cells, and pericytes. (B) The dot plot shows cell type–specific marker gene expression patterns that were used for population annotation and identification. (C) The feature plot visualizes the expression distribution of canonical marker genes across different cell populations. (D) The bar plot compares cellular composition percentages across different datasets and reveals notable heterogeneity in tumor microenvironment composition among patient cohorts. (E) The feature plot demonstrates that the 13‐gene signature score exhibits substantial heterogeneity across cell populations, with elevated activity particularly in proliferating OS cells, OS cells, and fibroblast populations. (F) The violin plot quantifies the signature score distribution across different cell types and confirms the highest expression in tumor cells and tumor‐associated stromal cells. (G) The correlation heatmap shows strong positive associations between the 13‐gene signature and pathways related to proliferation, metastasis, invasion, cell cycle regulation, and angiogenesis. (H) The multialgorithm pathway enrichment analysis demonstrates that high‐risk clusters exhibit significantly more upregulated pathways, while low‐risk clusters show a balanced or opposite pattern. (I) The comprehensive pathway analysis reveals that high‐risk groups are characterized by enrichment of malignant processes including metabolic reprogramming, MYC targets, mTORC1 signaling, PI3K–AKT–mTOR signaling, TNF*α*–NF*κ*B signaling, hypoxia response, E2F targets, and G2M checkpoint activation. (J) The dot plot displays the top differentially expressed genes between risk groups, including SPP1, MMP13, SERPINE1, TIMP1, IBSP, IGFBP7, ACTA2, and B2M, which are predominantly involved in extracellular matrix remodeling, osteogenesis, and tumor microenvironment regulation.

To investigate the cellular origin and microenvironmental interaction mechanisms of the 13‐gene signature, we analyzed publicly available single‐cell transcriptomic data from OS patients. After batch effect correction, we identified major cell populations including OS cells, proliferating OS cells, osteoblasts, fibroblasts, monocytes, myeloid cells, T/NK cells, TAMs, B cells, endothelial cells, and pericytes, comprising 97,864 cells from 17 samples (Figure 4A). The distinct cell type–specific expression patterns are displayed in Figure 4B,C. Cellular composition analysis revealed notable differences between datasets, suggesting heterogeneity in TME composition across patient cohorts (Figure 4D). The 13‐gene signature score exhibited substantial heterogeneity across cell populations, with elevated activity in proliferating OS cells, OS cells, and fibroblast populations (Figure 4E,F), indicating that the signature primarily reflects biological processes associated with tumor cells and tumor‐associated stromal cells. Pathway analysis revealed that the 13‐gene signature showed strong positive correlations with proliferation, metastasis, invasion, cell cycle, and angiogenesis pathways (Figure 4G). Multialgorithm validation demonstrated that high‐risk clusters exhibited significantly more upregulated pathways, while low‐risk clusters showed a balanced or opposite pattern (Figure 4H). High‐risk groups were characterized by enrichment of malignant processes including metabolic reprogramming, proliferation and growth (MYC targets, mTORC1 signaling, and PI3K–AKT–mTOR signaling), inflammatory and microenvironmental regulation (TNF*α*–NF*κ*B signaling and hypoxia response), and cell cycle progression (E2F targets and G2M checkpoint), which together drive OS progression, invasion, metastasis, and TME remodeling (Figure 4I). DEA identified key molecular drivers, with the top DEGs including SPP1, MMP13, SERPINE1, TIMP1, IBSP, IGFBP7, ACTA2, and B2M (Figure 4J). These genes are predominantly involved in extracellular matrix remodeling, osteogenesis, and TME regulation, providing mechanistic insights into the biological basis of the 13‐gene signature in OS.

### 3.5. Differentiation States and Cell–Cell Communication Patterns Associated With the 13‐Gene Signature

**Figure 5:**
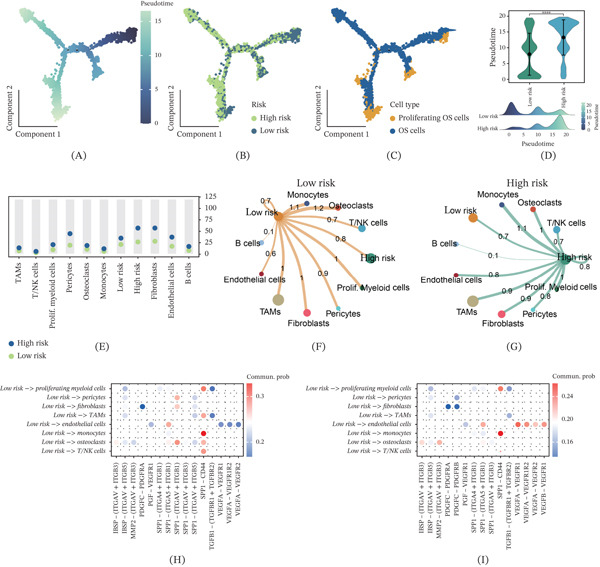
Differentiation states and cell–cell communication patterns associated with the 13‐gene signature. (A) The pseudotime trajectory analysis plot shows that high‐risk and low‐risk cells occupy distinct regions along the differentiation trajectory when colored by risk status. (B) The pseudotime trajectory plot colored by pseudotime values illustrates the progression of cellular differentiation states across the osteosarcoma (OS) cell populations. (C) The trajectory plot overlaying proliferating OS cells and OS cells demonstrates that these populations show spatial separation along the differentiation path. (D) The violin plot quantifies that high‐risk cells exhibit significantly higher pseudotime values compared to low‐risk cells (*p* < 0.001), suggesting enhanced dedifferentiation or stem cell–like characteristics. (E) The bar plot shows that high‐risk samples exhibit more ligand–receptor pairs across multiple cell type interactions, indicating increased complexity of communication networks. (F) The cell–cell communication network diagram reveals that the low‐risk group displays high‐intensity and focused communication patterns concentrated in specific signaling pathways. (G) The cell–cell communication network diagram demonstrates that the high‐risk group shows more extensive but relatively dispersed communication networks with reduced individual pathway strength. (H) The ligand–receptor interaction heatmap confirms that the low‐risk group maintains high communication probability concentrated in specific signaling pathways. (I) The ligand–receptor interaction heatmap reveals that the high‐risk group exhibits broader but more scattered ligand–receptor interaction patterns involving extracellular matrix remodeling, growth factor signaling, and immune regulation pathways.

To investigate the relationship between the 13‐gene signature and cellular differentiation dynamics, we performed pseudotime trajectory analysis. The analysis revealed that high‐risk and low‐risk cells occupied distinct regions along the differentiation trajectory (Figure 5A,B), with proliferating OS cells and OS cells showing spatial separation on the pseudotime trajectory (Figure 5C). Quantitative analysis demonstrated that high‐risk cells exhibited significantly higher pseudotime values compared to low‐risk cells (*p* < 0.001, Figure 5D), with high‐risk cells enriched at later pseudotime stages, suggesting enhanced dedifferentiation or stem cell–like characteristics. Cell–cell communication analysis showed that high‐risk samples exhibited more ligand–receptor pairs across multiple cell type interactions (Figure 5E), indicating increased complexity of communication networks. Network analysis revealed that the low‐risk group displayed high‐intensity and focused communication patterns (Figure 5F), whereas the high‐risk group showed more extensive but relatively dispersed communication networks with reduced individual pathway strength (Figure 5G). Ligand–receptor pair analysis further confirmed that the low‐risk group maintained high communication probability concentrated in specific signaling pathways (Figure 5H), while the high‐risk group exhibited broader but more scattered ligand–receptor interaction patterns (Figure 5I), involving extracellular matrix remodeling, growth factor signaling, and immune regulation pathways. These results demonstrate that the 13‐gene signature not only reflects tumor cell dedifferentiation characteristics but also captures the reorganization of cell–cell communication networks in the microenvironment, providing mechanistic insights into OS progression.

### 3.6. Spatial Transcriptomic Validation of the 13‐Gene Signature in Chemoresistant OS

**Figure 6:**
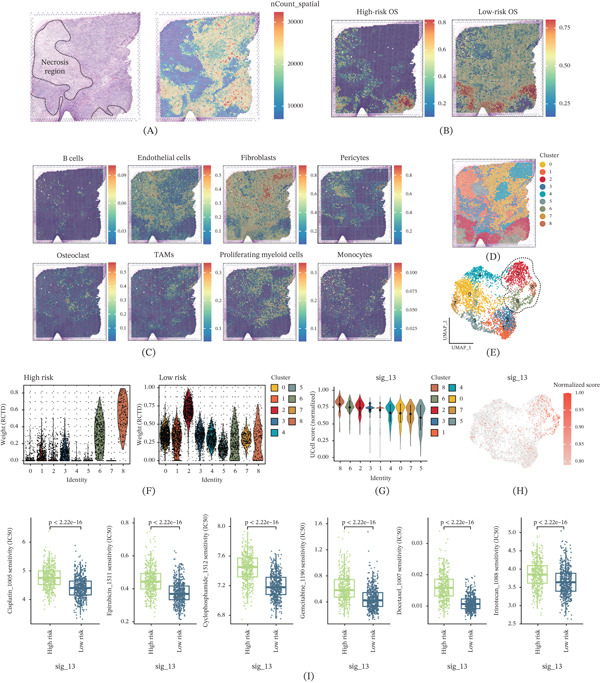
Spatial transcriptomic validation of the 13‐gene signature in chemoresistant osteosarcoma (OS). (A) The H&E staining image reveals prominent necrotic areas within the OS tissue section and provides histological context for spatial analysis. (B) The spatial mapping of RCTD deconvolution results reveals distinct spatial distributions of high‐risk and low‐risk OS cells, with high‐risk cells predominantly located at the necrotic periphery. (C) The spatial analysis shows that monocytes colocalize with high‐risk OS cells at the necrotic periphery, suggesting their involvement in immune regulation and chemoresistance mechanisms. (D) The unsupervised spatial clustering analysis identifies nine distinct spatial clusters with unique transcriptional profiles across the tissue section. (E) The spatial distribution map displays the localization of the nine identified clusters across the OS tissue. (F) The deconvolution weight analysis reveals that Clusters 2, 6, and 8 are predominantly composed of OS cells, with Cluster 2 showing low‐risk features and Clusters 6 and 8 exhibiting high‐risk characteristics that align with their location at the necrotic periphery. (G) The spatial visualization of 13‐gene signature scores demonstrates significant heterogeneity across clusters, with Clusters 6 and 8 displaying elevated scores and Cluster 2 showing lower scores. (H) The spatial UMAP mapping confirms the same expression pattern across Clusters 2, 6, and 8, thereby linking the signature to microenvironmental adaptation and chemoresistance. (I) The drug sensitivity analysis reveals significantly higher predicted IC50 values for all six chemotherapeutic drugs, including cisplatin, epirubicin, cyclophosphamide, gemcitabine, docetaxel, and irinotecan, in the high‐risk group, which confirms chemoresistance at the spatial level and consistency with clinical features.

To validate the clinical relevance of the 13‐gene signature at the spatial level, we performed spatial transcriptomic analysis on chemoresistant OS tissue. H&E staining revealed prominent necrotic areas (Figure 6A), with spatial transcriptomic data showing distinct transcriptional activity patterns across regions. RCTD deconvolution revealed distinct spatial distributions of high‐risk and low‐risk OS cells (Figure 6B). High‐risk OS cells were predominantly located at the necrotic periphery, forming a clear spatial boundary and suggesting enhanced adaptation of high‐risk cells to hypoxic microenvironments. Spatial analysis showed that monocytes colocalized with high‐risk OS cells at the necrotic periphery (Figure 6C), suggesting their involvement in immune regulation and chemoresistance. Unsupervised clustering identified nine spatial clusters (Figure 6D,E). Deconvolution weight revealed that Clusters 2, 6, and 8 were predominantly OS cells, with Cluster 2 showing low‐risk features and Clusters 6 and 8 exhibiting high‐risk characteristics (Figure 6F). This pattern aligned with tissue architecture, with Clusters 6 and 8 located at the necrotic periphery. Meanwhile, the 13‐gene signature score showed significant heterogeneity across clusters (Figure 6G). Clusters 6 and 8 displayed elevated sig_13 scores, while Cluster 2 showed lower scores, validating the signature’s effectiveness in distinguishing risk groups. Spatial UMAP mapping confirmed the same expression pattern in Clusters 2, 6, and 8 (Figure 6H), linking the signature to microenvironmental adaptation and chemoresistance. Drug sensitivity analysis revealed significantly higher predicted IC50 values for all six chemotherapeutic drugs (cisplatin, epirubicin, cyclophosphamide, gemcitabine, docetaxel, and irinotecan) in the high‐risk group (Figure 6I), confirming chemoresistance at the spatial level and consistency with clinical features. These results spatially validated the 13‐gene signature, revealing that high‐risk tumor cells localize to the hypoxic necrotic periphery and exhibit chemoresistance, providing mechanistic insights into OS treatment resistance.

## 4. Discussion

Chemotherapy resistance remains the primary barrier to improving long‐term survival in OS, yet the molecular mechanisms driving this resistance remain incompletely understood. In this study, we integrated transcriptome sequencing of chemotherapy‐resistant and sensitive OS samples with WGCNA to identify a 13‐gene signature that robustly predicts both chemotherapy response and clinical prognosis. This model demonstrated consistent prognostic value across multiple independent cohorts, effectively stratifying patients into high‐risk and low‐risk groups with significantly divergent survival outcomes. The signature’s ability to distinguish resistant from sensitive patients in our institutional cohort, combined with its validation in external datasets, supports its potential as a clinically applicable biomarker for treatment stratification.

The biological underpinnings of this signature reveal critical insights into the aggressive nature of high‐risk OS [56, 57]. Functional enrichment analysis demonstrated that high‐risk tumors exhibit pronounced activation of cell cycle regulation, DNA replication, and chromosome segregation pathways, accompanied by aberrant p53 signaling [58–61]. These features collectively indicate heightened proliferative capacity and genomic instability, hallmarks of aggressive malignancy that confer therapeutic resistance [62–66]. At the genomic level, high‐risk patients displayed elevated TMB with frequent mutations in TP53 and MUC16, suggesting that checkpoint dysfunction and genomic chaos may drive both intrinsic drug resistance and immune evasion [53, 60, 67–73]. While high mutation burden typically correlates with immunotherapy responsiveness, our findings paradoxically revealed a cold tumor phenotype in the high‐risk group, characterized by elevated TIDE scores indicating active immune escape mechanisms. This disconnect between mutational load and immune reactivity highlights the complex immunobiology of OS, where the suppressive TME effectively neutralizes potential immunogenicity [74–76].

The therapeutic implications of our risk stratification extend beyond prognosis to direct clinical decision‐making. Drug sensitivity analysis revealed that high‐risk patients exhibited marked resistance to first‐line chemotherapeutic agents including doxorubicin and methotrexate [77, 78], aligning with our clinical observations and suggesting that alternative therapeutic strategies should be considered for this population [79]. The identification of these resistant phenotypes prior to treatment initiation could enable more personalized approaches, potentially sparing patients from ineffective standard regimens and associated toxicities while directing them toward investigational therapies or combination strategies targeting specific resistance mechanisms. Furthermore, the extensive tumor–stromal crosstalk identified in high‐risk samples suggests that stromal‐disrupting strategies, such as fibroblast activation protein (FAP)–targeting agents, TGF‐*β* signaling inhibitors, or cancer‐associated fibroblast (CAF) normalization approaches, represent promising avenues to overcome the stromal barrier that physically and biochemically shields high‐risk tumor cells from chemotherapeutic assault. Future studies integrating these microenvironment‐targeted strategies with conventional chemotherapy may hold particular promise for improving outcomes in high‐risk OS patients.

Single‐cell and spatial transcriptomic analyses provided crucial mechanistic insights into how this signature operates within the tumor ecosystem [47, 80]. We found that the 13‐gene signature was predominantly expressed in tumor cells and fibroblasts, with high‐risk cells demonstrating dedifferentiated, stem‐like characteristics associated with enhanced malignant potential. Pseudotime trajectory analysis positioned these high‐risk cells at later differentiation stages, suggesting that chemotherapy resistance emerges through cellular plasticity and the acquisition of progenitor‐like properties [81–84]. Perhaps most significantly, cell–cell communication analysis revealed extensive crosstalk between the high‐risk tumor cells and the surrounding stromal microenvironment, particularly with fibroblasts and mesenchymal stem cells [85–87]. This tumor–stroma interaction network appeared more dispersed yet pervasive in high‐risk samples, potentially establishing a protective niche that physically and biochemically shields tumor cells from chemotherapeutic assault. The spatial validation confirmed these observations, showing that high‐risk cells concentrated at hypoxic necrotic peripheries where they colocalized with immunosuppressive monocyte populations, creating microenvironmental conditions conducive to survival under therapeutic stress.

Several limitations warrant consideration. The retrospective design and modest sample size of our institutional cohort necessitate prospective validation in larger, multicenter studies to confirm generalizability. Additionally, while our multiomics approach identified robust associations, the specific molecular mechanisms through which individual signature genes mediate resistance require functional validation through in vitro and in vivo experimentation. The precise signaling pathways connecting genomic alterations, transcriptional programs, and microenvironmental remodeling remain to be fully elucidated.

In summary, this study establishes a clinically relevant 13‐gene signature that integrates chemotherapy resistance prediction with prognostic stratification in OS. By connecting transcriptional profiles to genomic instability, immune evasion, and tumor–stroma interactions, our findings illuminate the multidimensional nature of therapeutic resistance and identify potential intervention points. This signature offers a foundation for precision oncology approaches in OS, enabling risk‐adapted treatment selection and highlighting the therapeutic potential of targeting TME crosstalk to overcome chemotherapy resistance.

## 5. Conclusion

In summary, we have developed and validated a 13‐gene prognostic signature that robustly predicts chemotherapy response and survival in OS. Through multiscale integrative analysis spanning bulk transcriptomics, single‐cell profiling, and spatial transcriptomics, we have elucidated the biological underpinnings of high‐risk disease: proliferative hyperactivation, genomic instability, immunosuppressive microenvironment remodeling, and tumor–stromal symbiosis. These findings provide a mechanistic framework for understanding chemoresistance in OS and identify actionable therapeutic targets, including stromal modulation and immune contexture reprogramming. The 13‐gene signature represents a promising precision medicine tool with potential to transform risk stratification and therapeutic decision‐making in this challenging malignancy, ultimately improving outcomes for the substantial subset of patients currently refractory to standard treatment protocols.

## Author Contributions

Zhuoran Tang, Xuanhong Jin, Haowei Wang, and Jianfei Tang conceived and supervised the study. Xiyu Yang and Xiaoming Lu designed the study and performed the bioinformatic analyses, including bulk RNA‐seq processing, WGCNA, prognostic modeling, and multicohort validation. Zhiqiang Gao and Wentao Lv collected clinical samples and conducted RNA extraction and transcriptome sequencing. Shenghuang Shen and Sijia Liu performed somatic mutation analysis, drug sensitivity prediction, and immune infiltration analysis. Zetong Li, Ruixuan Liu, Xinyao Wang, Yao Shen, and Danlei Yu conducted scRNA‐seq analysis, spatial transcriptomic integration, and cell–cell communication analysis. Xiyu Yang drafted the manuscript. Zhuoran Tang, Xuanhong Jin, Haowei Wang, and Jianfei Tang critically revised the manuscript for important intellectual content. Xiyu Yang, Xiaoming Lu, Zhiqiang Gao, Wentao Lv, and Shenghuang Shen contributed equally to this work.

## Funding

This study was supported by the High‐level Talent Introduction Project of Nantong University (grant no. 135424631064).

## Disclosure

All authors reviewed and approved the final manuscript.

## Ethics Statement

The collection of human tissue samples and related experiments in this study have been approved by the Ethics Committee of Shanghai Jiao Tong University (ethical approval code: 2025KS840).

## Conflicts of Interest

The authors declare no conflicts of interest.

## Data Availability

The data that support the findings of this study are available from the corresponding authors upon reasonable request.
